# Epirubicin-induced Kounis syndrome

**DOI:** 10.1186/s12872-021-01936-4

**Published:** 2021-03-12

**Authors:** Hui-zhu Liang, Hong Zhao, Jian Gao, Cheng-fu Cao, Wei-min Wang

**Affiliations:** 1 Department of Cardiology, Peking University People’s Hospital, Beijing, China; 2 Department of Radiology, Peking University People’s Hospital, Beijing, China

**Keywords:** Kounis syndrome, Epirubicin, Allergy, Hypovolemic shock, Acute coronary syndrome

## Abstract

**Background:**

Kounis syndrome is an acute coronary syndrome that appears in the setting of anaphylactic reaction or hypersensitivity. Many drugs and environmental exposures have been identified as potential offenders, and diagnosis and treatment can be challenging.

**Case presentation:**

A 62-year-old man with recurrent bladder cancer underwent an intra-iliac artery epirubicin injection. After the injection, he developed chest pain and a systemic allergic reaction, with electrocardiographic alterations and elevated troponin-I levels. Emergent coronary angiography showed right coronary artery spasm and no stenosis of the other coronary arteries. This reaction was considered compatible with an allergic coronary vasospasm. A diagnosis of Kounis syndrome was made.

**Conclusions:**

Kounis syndrome is common, but a prompt diagnosis is often not possible. ﻿This case is the first to suggest that an intraarterial epirubicin injection could potentially be one of its triggers. All physicians should be aware of the pathophysiology of this condition to better recognize it and start appropriate treatment; this will prevent aggravation of the vasospastic cardiac attacks and yield a better outcome.

## Background

The simultaneous occurrence of acute coronary syndrome with hypersensitivity reactions is named Kounis syndrome [1]. In 1950, Pfister first reported myocardial infarction and urticaria after penicillin treatment [2]. In 1991, Kounis and Zavras introduced the notion and pathophysiology of vasospastic angina and myocardial infarction related to allergies [3]. This entity was redefined as “acute coronary syndrome related to platelet and mast-cell activation in the course of hypersensitivity and allergic or anaphylactic events” [1]. It is triggered by inflammatory mediators, including histamine, neutral proteases, arachidonic acid products, platelet-activating factor, and a variety of cytokines and chemokines released following hypersensitivity and allergic activation [4]. These mediators can cause coronary vasospasm or atheromatous plaque erosion or rupture or even coronary thrombosis, resulting in myocardial infarction [5]. This syndrome is associated with serious morbidity and mortality, as it could be complicated by cardiac arrest or even death [6]. Although it is rarely reported, it is imperative to understand that Kounis syndrome is usually underdiagnosed [7].

Here, we report the first case of Kounis syndrome induced by an intra-iliac artery epirubicin injection in a bladder cancer patient.

## Case presentation

**Fig. 1:**
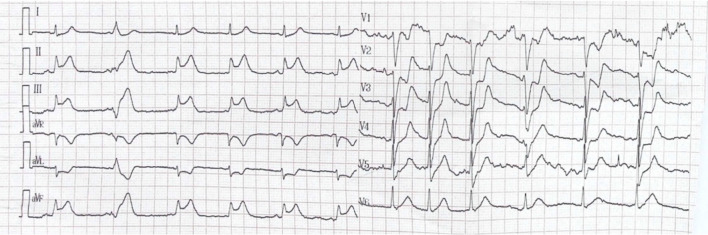
Electrocardiogram (ECG) at presentation: ST segment elevation of 3 mm in leads II, III and aVF and ST depression in leads I, and aVL, V1–V5

**Fig. 2:**
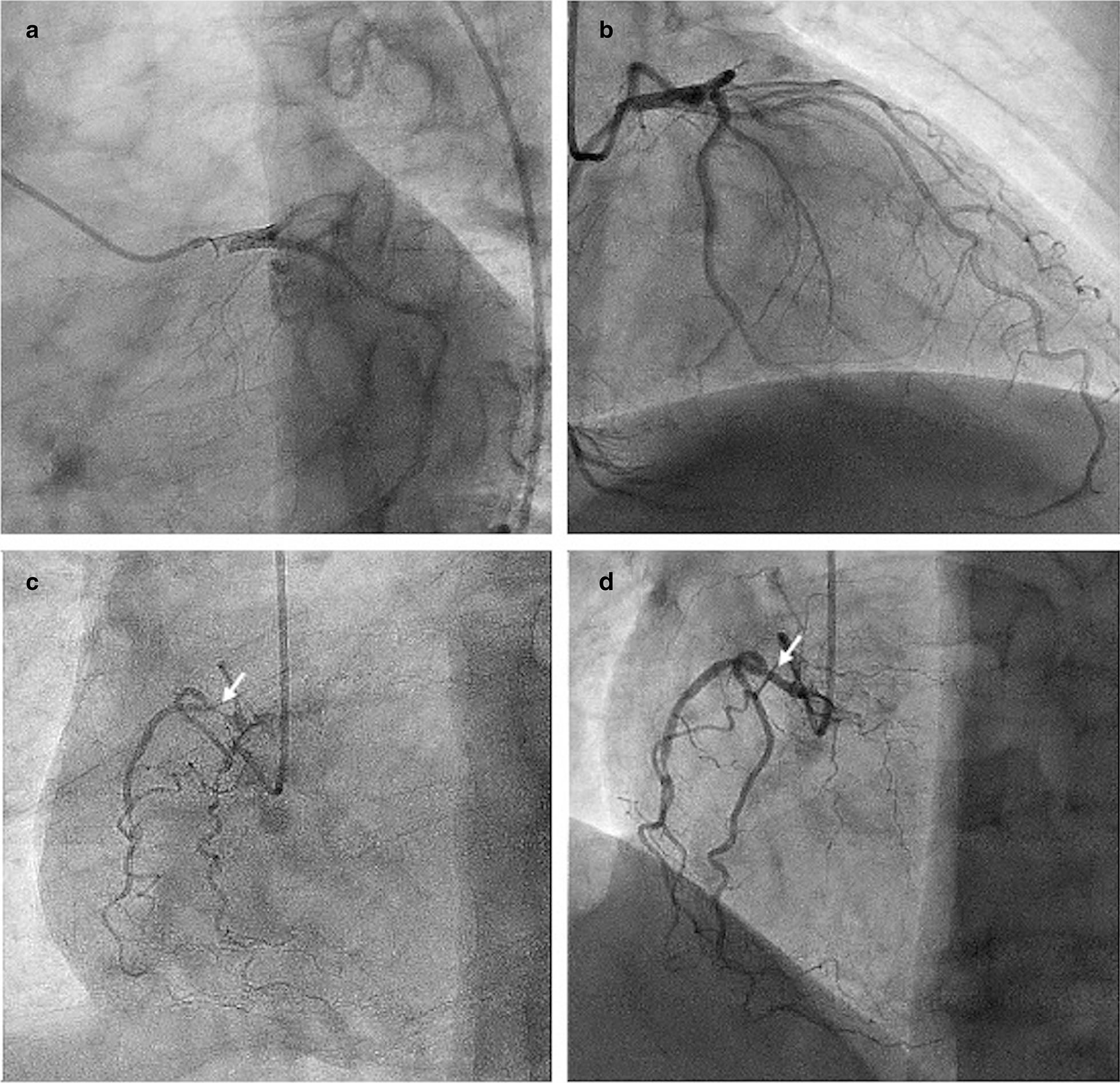
Coronary angiography: **a** Left main coronary artery (LMCA) reveals no significant disease; **b** left anterior descending branch coronary artery (LAD) and left circumflex coronary artery (LCX) showing no significant disease; **c** Observation of the right coronary artery (RCA) showing 90% stenosis in the proximal segment; **d** The stenosis of RCA was relieved after the intracoronary administration of nitrates

**Fig. 3:**
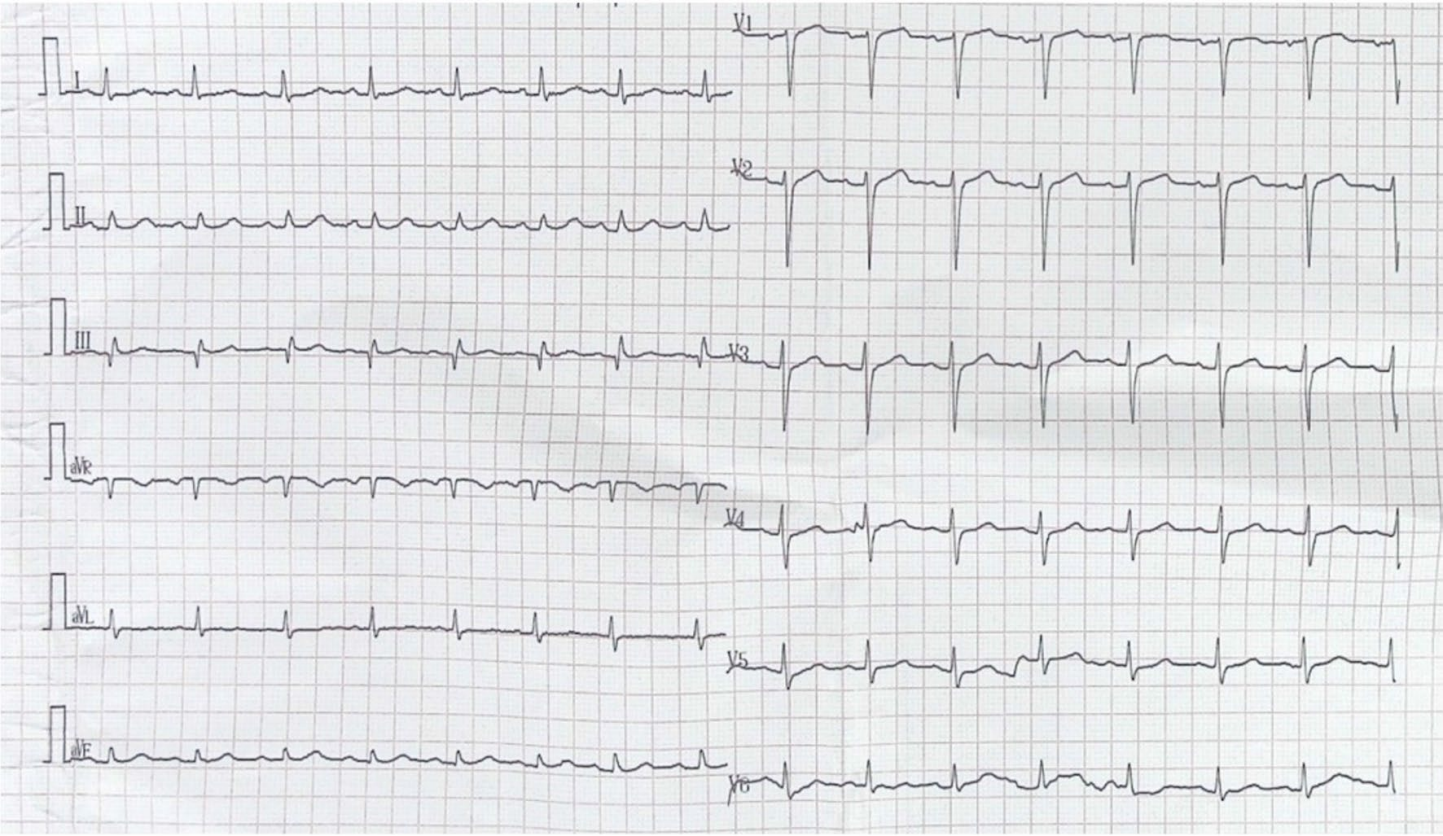
Electrocardiogram (ECG) after angiography: ST segment recovery

A 62-year-old man with recurrent bladder cancer was scheduled for intra-iliac artery chemotherapy. He was a smoker for more than 30 years and had a 10-year history of hypertension. No allergies were reported. There was no coronary artery disease in his family history. The preoperative echocardiogram showed no abnormalities and normal left ventricular ejection fraction. A percutaneous catheter system was set in the bilateral internal iliac arteries distal to the superior gluteal arteries by applying a modified Seldinger technique. During the operation, epirubicin (50 mg/m^2^) was dissolved in 50 ml of saline solution and then administered over 10 min. After injection, the patient suddenly developed severe dyspnea, chest pain, a red itchy rash on his face, subcutaneous edema, palpitation, diaphoresis and nausea. He progressively became hypotensive and eventually developed shock, and his blood pressure nadired at 52/48 mmHg. Corticosteroids and norepinephrine were administered for his allergic reaction. In addition, fluid resuscitation with 3L of crystalloids and boluses of dopamine was performed to keep the blood pressure at normal values. The first electrocardiogram (ECG) showed ST segment elevation of 3 mm in leads II, III and aVF and ST depression in leads I, aVL, and V1–V5 (Fig. 1). The patient was taken to the cardiac catheter laboratory for emergent angiography, which revealed 90% stenosis in the proximal segment of the right coronary artery (RCA); the remaining coronary vessels were normal. The RCA stenosis was relieved after administration of 200ug intracoronary nitroglycerin (Fig. 2). The ECG after angiography showed recovery of the ST elevation in the inferior leads (Fig. 3). The patient’s symptoms immediately resolved after coronary angiography, and his vitals stabilized.

The patient was monitored in the cardiac intensive care unit (CCU). His vital signs stabilized (temperature 37.1 °C, blood pressure 107/71 mmHg, heart rate 93 bpm, respiratory rate 22 breaths per minute). His initial ECG in the CCU showed normal sinus rhythm. Serial troponin-I monitoring showed a normal level (normal range 0.01–0.023 ng/mL) at the time of symptom onset, 0.056 ng/mL 5 h after onset, and a peak of 0.113 ng/mL at the 10th hour. The patient was treated with anti-ischemic treatment, including antiplatelet therapy (clopidogrel 75 mg), and nitrates were initiated after the coronary angiogram. During hospitalization, the patient remained free of chest pain, and he did not show any chest pain or systemic reactions again during the follow-up.

## Discussion and conclusion

Kounis syndrome, also called allergic angina, is caused by an anaphylactic or anaphylactoid insult. Acute coronary syndrome in Kounis syndrome may manifest as coronary spasm, acute myocardial infarction, or stent thrombosis [8]. A recent large epidemiological study in the USA demonstrated that the prevalence of Kounis syndrome was 1.1%, with a subsequent inpatient all-cause mortality rate of 7.0%. Compared to the non-Kounis acute coronary syndrome group, the Kounis syndrome group was older and had more males, more Caucasian patients, a longer duration of hospitalization and higher hospitalization charges. The rates of arrhythmias, cerebrovascular events and venous thromboembolisms were obviously higher in the Kounis syndrome group than in the non-Kounis syndrome group [9]. Any substance, disease entity or environmental exposure might be the causes of Kounis syndrome. Antibiotic and insect bites represent the most common triggers (27.4% and 23.4% respectively) [6]. Moreover, carboplatin [10], contrast media [11], isotretinoin [12], latex [13], and cobra bites [14] have also been identified as offenders. Therefore, an in-depth patient clinical history is mandatory for diagnosing Kounis syndrome. Laboratory (cardiac biomarkers, serum histamine, eosinophils or immunoglobulin E), electrocardiographic, echocardiographic and angiographic findings are valuable diagnostic tools [6].

Kounis syndrome is divided into three subtypes according to the condition of the coronary arteries: type 1, with normal vessels, where endothelial dysfunction causes allergic vasospastic angina; type 2, with preexisting coronary atheroma, where allergic reaction could lead to myocardial infarction (allergic myocardial infarction); and type 3, with previously treated coronary thrombosis, where recurrent thrombosis may occur. Kounis syndrome type 1 represents the vast majority of cases, with a good response to pharmacological therapy [6]. The clinical manifestations of Kounis syndrome may appear as hypersensitivities or allergic reactions in the early stage, followed by cardiac symptoms such as acute chest pain, palpitations, and dyspnea. Chest pain occurs in 86.8% of patients and represents the most common cardiac manifestation. Anaphylactic symptoms can emerge in approximately 53.0% of patients, however, 40% of patients with perioperative Kounis syndrome may not have an initial anaphylactic reaction [6, 15]. In Kounis type 1, approximately 2.3% of patients may develop shock [6].

There are no large, randomized data to guide the treatment of allergic angina pectoris, as most of the knowledge is based on individual cases reports. Nonetheless, the optimal clinical management of Kounis syndrome not only requires rapid diagnosis and decision-making but also depends on the syndrome subtype [1]. Treating Kounis syndrome patients according to a cardiovascular emergency therapeutic protocol might not be effective [16]. First, it is imperative to remove the potential allergen if feasible, and then the use of fluid resuscitation is particularly important in cases of anaphylactic shock. One should refrain from using medications that could exaggerate anaphylaxis and hypotension. For example, although vasospasm is commonly treated with vasodilators such as nitrates or calcium channel blockers, in Kounis syndrome, they may exacerbate hypotension [1]. Opiates such as morphine are very effective in managing acute coronary syndrome pain, but they have to be used cautiously in patients with Kounis syndrome, as they have been associated with mastocyte activation and worsening allergic symptoms [17]. One should also take into account the cardiac toxicities of the medication used to treat allergic reactions. For example, while intramuscular epinephrine is the key treatment for anaphylaxis, it may aggravate coronary spasm and ischemia or cause arrhythmias and QTc interval prolongation [18, 19].

This case represents a typical Kounis syndrome type 1 patient, characterized by coronary spasm in normal ﻿or nearly normal coronary arteries without predisposing factors for coronary artery disease. The vasospastic angina was likely induced by the rapid release of allergic mediators after epirubicin injection. Intra-iliac artery chemotherapy represents an important therapeutic modality for preventing recurrence and the growth of non-muscle invasive bladder carcinoma [20]. Epirubicin, a doxorubicin derivative, is rarely reported to be an allergen or cardiac toxicity [21]. To date, epirubicin had never been reported as a trigger for Kounis syndrome or any allergic reactions of the cardiovascular system. Anaphylaxis to gemcitabine [22] and cisplatin [23] has been reported, including acute myocardial infarction in Kounis syndrome. All anticancer drugs are able to induce allergic reactions and cardiohypersensitivity; consequently, the incidence of cardiovascular complications associated with cancer therapy is increasing and the treatment of malignant and cardiovascular diseases has become closely associated [24]. The need to incorporate several tests, measures, and evaluations before, during and long after chemotherapy to monitor for cardiac adverse events should be emphasized. It is critical to foster collaboration between cardiologists, oncologists, immunologists, pathologists, allergists and other medical professionals associated with cancer to optimize the management of patients with Kounis syndrome and to avoid delaying cancer therapy.

Regardless of the allergenic substances involved or the effective anti-allergic treatment administered, the early diagnosis for this patient was mainly clinical and mostly based on signs and symptoms that indicated allergy and myocardial ischemia after being exposed to the therapeutic modality. Furthermore, the coronary angiogram results showed that severe spasm of the RCA was quite paradigmatic. In addition, it has been reported that vasospasm affects mainly the right coronary artery in Kounis syndrome for no reason. [25].

In summary, Kounis syndrome, or “allergic angina syndrome”, is a rare but likely underdiagnosed entity [1]. Intra-arterial epirubicin injection could be an allergen associated with Kounis syndrome. Hypotension and shock in Kounis syndrome should be managed by treating both the anaphylaxis and the coronary etiology. It is a real challenge for physicians to recognize this syndrome early and to start adequate treatment, which may be needed to avoid fatal complications and improve outcomes.

## Data Availability

All relevant data supporting the conclusions of this article are included within the article.

## Abbreviations

ECG: Electrocardiogram.
CCU: Cardiac intensive care unit
